# Influence of Immediate Dentin Sealing on Bond Strength of Resin-Based CAD/CAM Restoratives to Dentin: A Systematic Review of In Vitro Studies

**DOI:** 10.3390/biomimetics9050267

**Published:** 2024-04-28

**Authors:** Iliana Antoniou, Petros Mourouzis, Dimitrios Dionysopoulos, Panagiotis Pandoleon, Kosmas Tolidis

**Affiliations:** 1 Department of Operative Dentistry, Faculty of Dentistry, School of Health Sciences, Aristotle University of Thessaloniki, 54124 Thessaloniki, Greece; ilianaantoniou.p@gmail.com (I.A.); pmourouzis@dent.auth.gr (P.M.); ktolidis@dent.auth.gr (K.T.); 2 Department of Prosthodontics, Faculty of Dentistry, School of Health Sciences, Aristotle University of Thessaloniki, 54124 Thessaloniki, Greece; ppandoleon@gmail.com

**Keywords:** bond strength, CAD/CAM, chairside materials, composites, dentin, immediate dentin sealing

## Abstract

Immediate dentin sealing (IDS) is a method of improving the bond strength of indirect dental restorative materials to dentin and belongs to the biomimetic protocols of contemporary dentistry. The purpose of this systematic review was to evaluate the effect of IDS on the bond strength of resin-based CAD/CAM materials to dentin. PubMed and MEDLINE, Scopus, and the Web of Science were searched by two individual researchers, namely for studies that have been published in English between 1 January 2005 and 31 December 2023 in accordance with the Preferred Reporting Items for Systematic Reviews and Meta-Analyses (PRISMA) statement. The inclusion criteria encompassed articles related to in vitro studies, measuring the bond strength through microtensile bond strength (μ-TBS), micro-shear bond strength (μ-SBS), tensile bond strength (TBS) or shear bond strength (SBS) tests after the use of the IDS technique. The included restorative materials comprised resin-based CAD/CAM materials bonded to dentin. A total of 1821 studies were identified, of which 7 met the inclusion criteria. A meta-analysis was not deemed appropriate due to the high level of diversity inthe publications and techniques. The use of IDS yielded higher bond strength outcomesin various experimental conditions and resin-based CAD/CAM materials. Overall, IDS in CAD/CAM restorations may contribute to better clinical outcomesand improved restoration longevity due to this property.

## 1. Introduction

The use of computer-aided design/computer-aided manufacturing (CAD/CAM) materials has grown significantly in the field of restorative dentistry [1]. This approach has come to replace traditional impression methods, stone models, and restorations made by technicians, aiming to minimize the human error inherent in analog procedures [2]. Inlay and onlay restorations can be fabricated using CAD/CAM blocks, offering a more minimally invasive approach than crowns. Advances in luting procedures have eliminated the need to emphasize the retention form of adhesive restorations [3]. Reducing the invasiveness of dental treatments by employing the minimal intervention approach offers benefits in clinical environments and represents a core component of advanced dental practice [4].

Digital technology has facilitated the use of resin composite blocks (i.e., Lava Ultimate (3M ESPE) and Cerasmart (GC Dental Products)), polymer-infiltrated ceramic network (PICN) materials (i.e., Enamic (VITA Zahnfabrik)) and filler press and monomer infiltration (FMPI) materials (i.e., Katana Avencia (Kuraray Noritake)) [5,6]. PICN as well as FMPI materials are polymerized under high pressure and high temperatures. Consequently, their manufacturing process leads to greater mechanical and biological properties than traditionally polymerized resin composites [5,6,7]. Furthermore, these composite resin blocks have good machinability as well as increased resistance to fractures, which are attributed to a low elastic modulus near dentin tissue [5,8,9,10,11,12]. Moreover, they are easier to mill, can be repaired and exhibit less marginal chipping and less wear of the antagonist teeth compared with glass ceramics [10,13].

The focus of the present research was on resin-based CAD/CAM restorative materials. Nevertheless, the developer of Lava Ultimate (3M ESPE) removed the crown indication a few years ago due to an allegedly significant debonding rate [6]. The endurance of a dental restoration is partially determined by its adhesive capability, a quality often assessed through bond strength testing. Clinicians have traditionally turned to laboratory assessments to choose adhesive systems for their routine procedures. Though the reliability of bond strength assessments for predicting the clinical efficacy of dental adhesives remains uncertain, there is an indication that specific categories of laboratory test outcomes can offer understanding ofclinical functionality [14,15].

Regarding the investigation of bonding on tooth substrate and the dissection of the structure of adhesions as a whole, there are two interfaces: dentin to resin luting cement and resin luting cement to CAD/CAM material [16]. From now on, in this systematic review, the phrase “structure of adhesion” will be used to describe the specimens, including dentin, the dentin coating, adhesive cement, and CAD/CAM material.

Many different surface treatment procedures for each CAD/CAM material surface have been suggested to reinforce the resin cement-to-block interface [17,18]. The most widely used techniques for creating micromechanical retention are hydrofluoric acid etching and sandblasting with aluminum oxide (Al_2_O_3_) particles [19,20,21,22], along with further chemical adhesion through silanes and active monomers [21,22]. Some studies investigating the shear bond strength (SBS) of resin-based CAD/CAM materials to resin composites showed there lative significance of chemical and micromechanical adhesion [23,24]. For the aforementioned reasons, the IDS technique belongs to the bond-maximizing protocols of biomimetic dentistry.

Concerning the dentin interface of the structure of adhesion, before the cementation of a restoration, the prepared dentin should be covered with a provisional filling [25]. Nevertheless, postoperative and pulpal sensitivity are frequently caused by the provisional filling material’s poor sealing performance and durability [26]. The clinical procedure called delayed dentin sealing (DDS) involves applying the dentin adhesive just before cementing the restoration in a return appointment [27]. A resin coating technique was first developed in the 1990s, aiming to minimize such issues through immediate application of the adhesive agent and flowable resin composite on the freshly cut dentin surface [28]. The immediate dentin sealing (IDS) technique involves applying the dental bonding agent directly onto freshly prepared dentin before making an impression [29]. Magne et al. in their research advocated for an IDS technique analogous to the resin coating technique [26,30]. Both the coating and immediate sealing techniques produce a new substrate: a hybrid layer acting as an integral barrier on the prepared dental tissue [27,31]. According to previous research, IDS prevents contamination of the prepared dentin, and the collagen fibers are shielded from collapse as the hybrid layer is protected. This leads to a greater bond strength and improved mechanical properties for indirect tooth restorations [32,33,34,35,36], enhancing cavity adaptation and marginal sealing as well as lowering the patient’s sensitivity after the luting appointment. Additionally, the IDS technique contributes to protection of the dentin-pulp complex as well as increasing the bond strength, as implied by several studies [37,38,39].

The purpose of this systematic review was to evaluate the impact of the IDS technique on the bond strength between resin-based CAD/CAM restorative materials and dentin, as well as the factors that may influence the bonding efficacy. The novelty of this systematic review compared with previous reviews which investigated this topicis the analysis of the effect of the IDS technique only on resin-based CAD/CAM materials, avoiding the factor of the type of material, which may increase the risk of bias in the study. We investigate the effect of the IDS technique on the bond strength exclusively, as these materials account for their unique composition, surface characteristics and adhesive properties. This specificity enhances the relevance and applicability of the findings to contemporary dental practices utilizing resin-based CAD/CAM materials, informs clinical decision making and improves the predictability of treatment outcomes in everyday practice.

Our investigation addresses a significant gap in the current literature, as previous studies primarily focused on the bond strength of CAD/CAM restoratives without considering the impact of the composition of the materials. By specifically examining the effect of the IDS technique, we aimed to provide valuable insights into optimizing bonding protocols and improving the longevity of resin-based restorations. Furthermore, our research offers a comprehensive analysis by exploring a range of parameters, including different types of resin-based CAD/CAM restoratives, luting agents, adhesive agents, surface pretreatments, temporary restorations and variations in IDS application techniques. This multifaceted approach allows us to elucidate the nuanced interactions between IDS and resin-based restoratives, paving the way for tailored bonding strategies that account for individual patient needs and clinical scenarios. The research hypothesis was that the IDS technique would exhibit a positive influence on the bond strength of resin-based CAD/CAM materials.

## 2. Materials and Methods

### 2.1. Focused Review Question

This systematic review was conducted following the Preferred Reporting Items for Systematic Reviews and Meta-Analyses (PRISMA) statement [40]. The scientific inquiry or question of this systematic review was “Does immediate dentin sealing technique have a positive influence on the bond strength of resin-based CAD/CAM materials?” The PICO question was structured as follows:

Population (P): Teeth that require resin-based CAD/CAM restorations;
Intervention (I): The IDS technique;
Comparison (C): With or without the IDS technique;
Outcome (O): Bond strength;
Statistical analysis: Qualitative analysis of in vitro studies.

### 2.2. Identification Screening and Eligibility of the Included Studies

#### 2.2.1. Literature Search Strategies

Three electronic databases were consulted during the electronic search, namely PubMed and MEDLINE, Scopus and the Web of Science for studies published between 1 January 2005 and 31 December 2023. A search strategy was followed for each database in four levels: level 1, considering the type of material put to the test; level 2, considering the type of test evaluating the bond strength; level 3, considering the bond strength testing with dentin; and level 4, considering the implementation of immediate dentin sealing protocols. The search strategy included the terms (MeSH) in the following scheme:

“Bonding” AND “CAD CAM” AND “resin”;
“Adhesion” AND “CAD CAM” AND “resin”;
“Bonding” AND “CAD CAM” AND “resin” AND “tooth”;
“Bonding” AND “CAD CAM” AND “resin” AND “dentin”;
“Adhesion” AND “CAD CAM” AND “resin” AND “dentin”.

#### 2.2.2. Inclusion and Exclusion Criteria

The inclusion criteria encompassed articles published in English related to in vitro studies measuring the bond strength through micro tensile bond strength (μ-TBS), micro-shear bond strength (μ-SBS), tensile bond strength (TBS) or shear bond strength (SBS) tests. The materials included were resin-based CAD/CAM materials bonded to dentin, having applied a protocol for a dentin coating technique. All selected publications’ titles and abstracts underwent meticulous processing to filter out content that was not relevant to this review. Only relevant data were collected from studies where bond strength testing was conducted on resin-based CAD/CAM restorative materials. Studies not including bonding tests were excluded. Studies with samples submitted to 4 point bending tests or used for the determination of the mini-interfacial fracture toughness (mini-iFT) were also excluded. Additionally, studies related to temporary CAD/CAM materials, glass ceramic CAD/CAM materials, CAD/CAM milled anatomical post and cores, CAD/CAM tooth-colored implant custom abutments, adhesion of CAD/CAM customized orthodontic brackets, CAD/CAM acrylic denture bases and experiments related to the repair bond strength of CAD/CAM materials were excluded.

The full-text article was reviewed if it was not possible to accurately discern the paper’s focus from the title or abstract. Initially, titles and abstracts were assessed by two independent researchers (I.A. and D.D.). Full texts were selected for both reading and eventual inclusion based on reference to the inclusion and exclusion requirements. A third researcher (P.M.) evaluated all discrepancies between the two researchers’ decisions, and through constructive dialogue, a consensus was reached. From all selected publications, the following determinants were collected and then analyzed: authors and years, dentin sealing protocols, types of CAD/CAM materials, adhesive procedures of the specimens, luting procedures, site of failure, type of failure, and evaluation methods.

### 2.3. Quality Assessment: Risk of Bias

The risk of bias of each selected paper was individually assessed by two researchers (I.A. and D.D.) employing the Risk of Bias tool for Pre-Clinical Dental Material Research (RoBDEMAT) [41,42,43]. The RoBDEMAT tool evaluates the quality of research on dental laboratory materials by assessing nine components pertaining to various sources of bias across four domains: bias associated with planning and allocation (D1), sample preparation (D2), outcome evaluation (D3), and data processing and outcome reporting (D4). The reviewers complied with the tool’s guidelines for responding to signaling questions, categorizing their answers as “sufficiently reported or adequate”, “insufficiently reported”, “not reported or not adequate” or “not applicable”. The quality assessment information is reported in Table 1. A review of these articles showed that the field of study targeted in this systematic review has significantly increased during the last eight years.

## 3. Results

**Figure 1:**
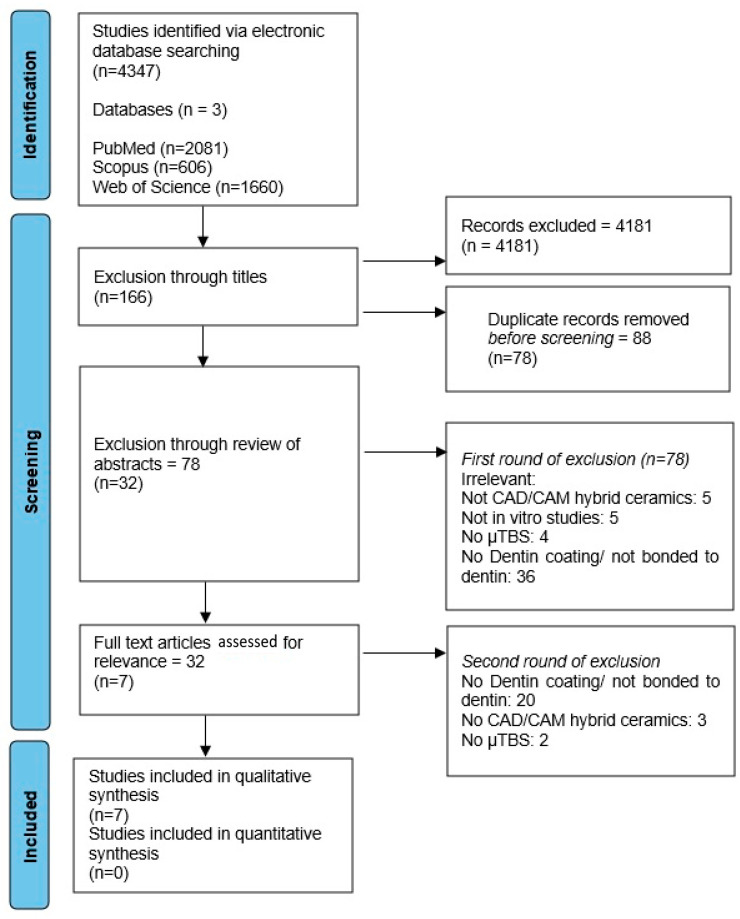
PRISMA flow diagram of the literature research and the inclusion and selection process. PRISMA =Preferred Reporting Items for Systematic Reviews and Meta-Analyses.

A literature search identified 4347 studies, with 2081 from PubMed and MEDLINE, 606 from Scopus, and 1660 from the Web of Science. After removing duplicates and reviewing their titles, 166 studies were selected for summary review. Upon examining the abstracts of these 166 articles, 32 were found to meet the inclusion criteria, and their full-text articles were assessed. However, 25 studies were subsequently excluded from the systematic review as they no longer satisfied the inclusion criteria. In total, the systematic review encompassed sevenin vitro studies [44,45,46,47,48,49,50]. The PRISMA flow methodology describing the search strategy is presented in Figure 1. The information and experimental parameters recorded for the studies included are presented in Table A1 and Table A2 (Appendix A). A meta-analysis was not deemed appropriate due to the high level of diversity in the publications and techniques.

All seven studies underwent risk of bias analysis based on the criteria established by the RoBDEMAT tool, as presented in Table 1. All studies maintained identical experimental conditions across the groups, employed adequate and standardized testing procedures and outcomes, conducted statistical analysis and reported sufficient study outcomes [44,45,46,47,48,49,50]. However, only five studies [44,45,47,48,49] provided comprehensive details regarding the randomization process. Two studies did not reference any randomization of samples [46,50]. Although the five studies mentioned the random allocation of specimens, they did not offer specific information on this procedure. All studies except one [46] adequately presented the standardization of samples and materials. None of the studies reported any blinding procedures. Furthermore, all studies conducting μ-TBS tests had specific sample sizes [44,45,46,47,49,50], although no study reported any sample size standardization procedure, while the one study that applied SBS testing did not describe any sample size determination [48]. Additionally, one study lacked a proper control group by not including a no-IDS group [47].

Six out of seven papers used human dentin specimens [44,45,47,48,49,50], and one paper used bovine incisors [46]. All studies implemented immediate dentin sealing techniques, described in further detail in Table A1. Three studies [44,46,47] implemented some form of temporarization, proposing protocols equivalent to multiple-visit treatments. Four papers [44,45,47,48] used self-adhesive resin cement, among other applications.

In the current review, four papers studied resin CAD/CAM blocks of Grandio block (VOCO), Katana Avencia (Kuraray Noritake) and Cerasmart (GC) [44,45,46,49], while three studied resin CAD/CAM blocks of Lava Ultimate (3M, ESPE) [47,48,50]. One study included resin CAD/CAM blocks of Lava Ultimate (3M, ESPE) alongside resin-ceramic CAD/CAM blocks of Vita Enamic (VITA) [50]. All studies followed the manufacturers’ instructions. Table 1 and Table A1 present further information regarding the scientific protocols of the studies included in this systematic review.

In six of the included studies, the specimens were subjected to μ-TBS testing [44,45,46,47,49,50], while researchers Sag and Bektas conducted TBS testing [48]. Three studies implemented stimulated pulp chambers [44,47,48]. Three studies carried out aging procedures, including thermocycling [49], cyclic loading [50] and 6 months of distilled water storage [44]. Five studies conducted a failure mode analysis by means of scanning electron microscopy (SEM) [45,46,47,49,50], three of which also conducted Weibull analysis [45,46,50]. One study conducted Weibull analysis of bond strength data [44].

Additional information was sourced by means of dentin permeability reduction percentage result comparison [44], polymerization light irradiance measurements performed in the second study [45], attenuated total reflectance Fourier transform infrared spectroscopy (ATR-FTIR) analysis in the third study [46] as well as confocal laser scanning microscopy (CLSM) observation conducted in the seventh study [50].

Four of the included studies [44,45,48,50] concluded that dentin sealing techniques distributed significantly higher bond strength results in various experimental conditions and materials. The studies revealed that applying a resin coating strengthened the dentin interface. The coated layer assumed a role as a low elastic modulus layer, functioning as a stress alleviator [45,46,49,50]. The structure of the adhesion is enhanced by the sealing techniques [44,45,47,48,49,50]. A weaker bond was speculated for a self-adhesive resin cement containing silane compared with a self-adhesive with a separate ceramic primer and silane [45].

## 4. Discussion

According to the outcomes of the selected studies of this systematic review, the use of the IDS technique resulted in higher bond strengths across various experimental conditions and materials. Consequently, the research hypothesis of the study, which stated that the IDS technique would exhibit a positive influence on the bond strength of resin-based CAD/CAM materials, was accepted. The effectiveness of the method regarding the survival of the restorations was also supported by various clinical studies [51,52,53]. Also, it was found that the IDS technique may reduce the post-cementation sensitivity of the restorations, possibly due to better sealing of the dentinal tubules before restoration procedures [54,55].

Contrary to previous scientific evidence, Sag and Betkas et al. [48] found that the use of temporary light-cured resin cements may lead to reduced adhesive strength, which also aligns with Abdou et al. [46], who found that temporization with non-eugenol zinc oxide cement resulted in a lower bond strength, regardless of the removal method of the temporary cement. Furthermore, Gailani et al. [47], found that the application of light-cured resin temporary cement did not change the results for the IDS technique in their tested protocols.

The results of the study of Abo-Alazm and Safy [44] revealed that the IDS technique reinforced the bond strength significantly both with and without aging protocols for the two adhesive agents. It is presumed that freshly cut dentin, free from contamination, is the ideal substrate for bonding [27]. Stress-free bonding is advantageous in the IDS technique, in addition to allowing for the increase in bond development over a time interval of one week [56]. In this study, GLUMA Bond Universal showed significantly higher values compared with the iBOND self-etch adhesive in all experimental groups. This may be attributed to the 10-methacryloyloxydecyl dihydrogen phosphate (10-MDP) functional monomer in the GLUMA Bond Universal ingredients, forming a more stable dentin bond due to MDP-Ca salt deposition [57]. The IDS technique obtained higher μ-TBS results after 24 h as well as after 6 months of water storage. Nevertheless, both examined adhesives exhibited a decline in bond strength after a 6month aging process. The diminished values may be attributed to the deterioration of their chemical interaction [58]. Another factor contributing to this deterioration is the potential of phase separation due to the vapor pressure disparities between the acetone and water in the tested adhesives [59]. The presence of water contributes to the degradation of collagen fibrils as well as plasticization of the composite, precipitating the deterioration of the hybrid layer and consequently lowering the dentin bond strength over time [60].

Oda et al. [45] highlighted the notable impact of the resin coating, resin cement and curing mode. The IDS group distributed a higher bond strength compared with the non-coated group. When simulating clinical single-visit conditions, not removing the oxygen inhibition of the polymerization layer resulted in superior adhesion after 1 h of water storage [61]. Additionally, a self-adhesive resin cement with a separate ceramic primer and silane yielded higher bond strength results compared with a self-adhesive cement containing silane. It is presumed that this may be due to water absorption and dissolution of the cement prior to the luting procedures. The structure of the adhesion is enhanced by the coating technique, as well as the dual curing mode, which reveals the weak mechanical properties of the tested resin cement through the most common encounter of cohesive failure within the cement itself. Another significant finding of Oda et al. [45] was the correlation between the resin coating and curing mode. In self-curing mode, the non-coated specimens performed significantly worse than the resin-coated ones. A possible explanation of this phenomenon may be the fact that the cement–dentin interface becomes brittle due to water uptake from the dentinal tubules [62], as well as the slow curing process [63]. The resin coating ensures an adequate hybrid layer, resulting in better bonding strength regardless of the cement or the curing mode parameters.

In Abdou et al.’s investigation [46], IDS using a one-bottle adhesive also improved the bond strength. Several factors have been found to play a role in the interpretation of these results and influencing the structure of the adhesion interface. The difference in the elapsed time starting from the preparation of the tooth and ending at the bonding of the final restoration affects the resin coating layer affects the degree of conversion of the polymerization process and may cause degradation of the coating layer due to water sorption [64]. Two of the tested one-bottle adhesives in this study contained 10-MDP as a functional monomer and showed higher bond strengths, which can probably be attributed to the formation of a nano-layer(10-MDP-Ca) at the dentin interface. The third tested one-bottle adhesive contained the functional monomer glycerol phosphate dimethacrylate (GPDM), which penetrates deeply into exposed collagen, is highly demineralizing to dentin and forms a strong polymer network [65]. The above properties may explain the insignificant difference in bonding performance of this material regardless of the number of visits or applications of resin coatings. The GPDM-Ca salt being more susceptible to water degradation than 10-MDP-Ca may explain the lower bond strength and the greater frequency of pretesting failures in the multiple-visit group of the Optibond/NX3 cement. Previous research showed a positive influence on the stability of the monomer-calcium ionic bonds rendered by the hydrophobicity and the longest spacer chains of the 10-MDP [66]. On the contrary, GPDM having shorter and hydrophilic spacer chains might have reduced the bonding strength of this adhesive agent following aging [46].

The adhesive strength values were shown to be mainly material dependent. In Gailani et al.’s study [47], a three-step etch-and-rinse adhesive with a high filler load and high mechanical strength yielded the highest μ-TBS results. The outcomes indicated that IDS yielded better μ-TBS values than the DDS technique for most of the adhesives and resin cements, in agreement with most of the reviewed literature [29,50,67]. In this study [47], concerning the structure of adhesion, there was a high percentage of fractures in the block–cement interface. Cohesive failures in dentin and the block-to-cement interface were discarded to evaluate the mean of the adhesive and the mixed failures of dentin and cement separately. These groups accurately reflect the resistance of the adhesion to dentin. It was presumed that when this type of failure happens, the real values of the bond strength are higher than the adhesive values of the cement-to-block bond [68].

In the study by Sag and Bektas [48], the IDS groups exhibited significantly higher bond strengths compared with the DDS groups. The enhanced bond strengths of IDS might be related to the pre-polymerization of dentin bonding agents. Polymerizing the bonding agent at the same time as the resin cement when seating the restoration and applying pressure might cause the collapse of the unpolymerized hybrid layer in the dentin interface [69]. The use of the IDS technique directly forms an unforced dentin-adhesive layer on the tooth. This bond can remain strong over a period of one week [56]. All of the above seem to validate an increase in bond durability with the sealing technique. The bonding agent used in the aforementioned study [48], having a higher filler content, may reduce polymerization shrinkage and consequently increase the bond strength [70]. Also, the higher demineralization depth achieved using this adhesive system ameliorates micromechanical retention and strengthens chemical bonding [71]. It has been shown that gap formation may be avoided with the application of a flowable resin composite on the adhesive interface, thus resulting in improved bond strength values [72]. The self-adhesive cements yielded lower bond strength results, which may be due to their inability to remove the smear layer [73].

Rozan et al. [49] demonstrated that the use of RelyX Ultimate cement as well as G-CEM Link Force on pretreated CAD/CAM restorations and dentin created a low elastic modulus layer that acted as a stress breaker, yielding higher bond strength results [74]. Also, Panavia resin cement exhibited a higher bond strength for the resin-coated group and the highest bond strength values encountered for the two-step flow method. The bonding agent, being hydrophilic, is protected from percolation of water by the relatively hydrophobic and flowable resin composite layer of the dentin sealing, which acts as a physical barrier [75]. The Rely X Ultimate cement performed better than the other cements regarding the uncoated group, supposedly due to the separate light curing of the adhesive before bonding. Separate light polymerization of the adhesive contributes to gaining a higher degree of conversion [63]. The bond strength test results for the IDS specimens were influenced differently by the various resin cements. This implies that the resin coating may act as a shield to the dentin, averting the debonding or fracturing of the restoration.

Ishi et al. [50] reported that restorations using IDS tend to be significantly advantageous concerning intra-cavity bond strength as well as bond reliability. Implementation of Weibull analysis revealed useful information concerning the improved bond reliability [76]. The significance of the PF 10 level as a property surpasses that of the mean μ-TBS value, providing a more suitable reflection of clinical conditions [77]. In situations with a low probability of failure, the outcomes regarding bond reliability and restoration performance when employing the dentin coating technique tend to be markedly superior compared withthose without the sealing technique, irrespective of the CAD/CAM material type.

Regarding the structure of adhesion, in association with the material selection and during bond strength testing, fractures occurred either in the weakest or at the most damaged parts of the specimens [50]. Resin cements present a lower flexural strength than flowable composites, and consequently, fracturing often occurs at the cement. Also, a cement-bondedrestorative material with a high modulus of elasticity may be directly damaged during cyclic loading procedures, resulting in cohesive failures. Referring to the bond reliability, stress redistribution was experimentally investigated during vertical occlusal loading by using three finiteelement analyses (FEAs). It was observed that the stress distribution was better when there were lower elastic modulus values for the materials. It was presumed that apparent as well as unapparent destruction occurs within the resin cement during cyclic loading, causing deterioration of the bond, and consequently, the more elastic material has better performance, regardless of the use of a sealing technique. As the Weibull modulus value increased, so did the bond reliability. Thus, the failure behavior became more predictable.

## 5. Conclusions

According to the analysis of the outcomes of the selected studies of this systematic review, the use of the IDS technique yielded higher bond strength results in various experimental conditions and resin-based CAD/CAM materials. More in vitro studies are required to investigate the bond strength of the entire structure of the adhesion. Precise and meticulous specimen preparation protocols and study designs are necessary for investigating the bond strength of the entire structure of the adhesion. Additionally, thoroughly interpreted result analyses are required to draw conclusions on the advantageous combinations of materials and techniques. The question arises as to which combination of in vitro experiment variables retrieves the best clinically related results in terms of bond strength reliability when testing complete adhesive structure specimens.

## Figures and Tables

**Table 1:** Risk of Bias tool for Pre-Clinical Dental Material Research (RoBDEMAT) of the selected studies.

No.	Date and Author	1.1 Control Group	1.2 Randomization of Samples	1.3 Sample Size Rationale and Reporting	2.1 Standardization of Samples and Material	2.2 Identical Experimental Conditions across Groups	3.1 Adequate and Standardized Testing Procedures and Outcomes	3.2 Blinding of the Test Operator	4.1 Statistical Analysis	4.2 Reporting Study Outcomes
1	Abo-Azlam and Safy, 2022 [44]	S	I	S	S	S	S	I	S	S
2	Oda et al., 2022 [45]	S	I	S	S	S	S	I	S	S
3	Abdou et al., 2021 [46]	S	N	S	I	S	S	I	S	S
4	Gailani et al., 2021 [47]	N	I	S	S	S	S	I	S	S
5	Sag and Bektas, 2020 [48]	S	I	N	S	S	S	I	S	S
6	Rozan, et al., 2020 [49]	S	I	S	S	S	S	I	S	S
7	Ishi et al., 2017 [50]	S	N	S	S	S	S	I	S	S

N = not reported; S = sufficiently reported; I = insufficiently reported.

## Data Availability

This study does not report any data.

## Appendix A

**Table A1:** Overview of the dentin sealing protocols and methodologies used in the selected studies.

No.	Date and Authors	Dentin Specimens	Dentin Sealing Techniques						Ageing	SEM	Additional Information
			IDS (Bonding Agent)	IDS (Bonding Agent) Immediate CAD/CAM Block Cementation	IDS (Bonding Agent and Flowable Composite)	IDS with Provisional	DDS with Provisional	Uncoated, No IDS, Control			
1	Abo-Azlam and Safy, 2022 [44]	Human third molars, 50 dentin specimens, 600 grit SiC	-	-	-	1. iBOND (self-etch) 2. GLUMA Bond (Universal) 3. Temporary cement	1. After preparation temporary cement 2. Temporary restorative discs	-	6 month distilled water storage	-	μ-TBS + Weibull analysis + Simulated pulp pressure
2	Oda et al., 2022 [45]	Human molars, 40 dentin specimens, 600 grit SiC	-	-	1. Clearfill SE Bond 2 (primer) 2. Clearfill SE Bond2 (bond) 3. Clearfil Majesty ES Flow	-	-	YES	-	Failure type analysis No pretesting failures	μ-TBS + Polymerization + Light irradiance measurement (spectoradiometer) + Weibull analysis
3	Abdouet al., 2021 [46]	60 bovine incisors, 600 grit SiC	1. Clearfil Universal Bond Quick 2. Scotchbond Universal Adhesive 3. Optibond All in One				1. Single-visit: distilled water at 37 °C for 1 h 2. Multiple-visit: eugenol-free temporary cement	YES	-	Failure type analysis	μ-TBS + ATR-FTIR analysis + Weibull analysis
4	Gailani et al., 2021 [47]	108 human third molars, MOD cavities	-	(IDS1) 1. Adhesive applied on specimen 2. Immediate final cementation	-	(IDS2) 1. Adhesive, glycerin gel and curing 2. Provisional restoration	(DDS) 1. Adhesive, provisional restoration 2. Two weeks simulated pulp pressure	-	-	Failure type analysis Pretesting failures included	μ-TBS + 9 different adhesives + Simulated pulp chamber
5	Sag and Bektas, 2020 [48]	120 human molars	-	-	1. Clearfil SE Bond 2. Filtek Ultimate Flowable	-	-	YES	-	No failure type analysis	SBS + Simulated pulp chamber
6	Rozanet al., 2020 [49]	72 human third molars, MOD cavities	G-Premio Bond	-	1. Clearfil SE Bond 2 2. Clearfil Majesty ES Flow	-	-	YES	Thermocycling for 5000 cycles (5–55 °C)	Failure type analysis	μ-TBS + CLSM observation
7	Ishi et al., 2017 [50]	24 mandibular first molars, MOD cavities, 100 μm grit diamond bur	-	-	1. Scotchbond Universal Adhesive 2. Filtek Supreme Ultra Flowable	-	-	YES	Cyclic loading stress	Failure type analysis No pretesting failures	μ-TBS + Weibull analysis

**Table A2:** The CAD/CAM materials, surface treatments, adhesive systems and luting cements used in the bonding procedures.

No.	Date and Author	CAD/CAM Material	Surface Treatment to CAD/CAM Blocks			Adhesive Agent	Luting Agent
			Sandblasting	Acid Etching	Silane		
1	Abo-Azlam and Safy, 2022 [44]	Grandio Block, VOCO	50 μm Al_2_O_3_ 10 s	-	Silane coupling agent	-	RelyXUnicem
2	Oda et al., 2022 [45]	Katana Avencia, Kuraray Noritake	50 μm Al_2_O_3_ 20 s	35% phosphoric acid 5 s	Silane coupling agent	Clearfil SE Βond 2 (self-etch)	1. Panavia SA Cement Plus 2. Panavia SA Cement Universal
3	Abdou et al., 2021 [46]	Katana Avencia, Kuraray Noritake	50 μm Al_2_O_3_ 60 s	1. No etching 2. No etching 3. 37.5% phosphoric acid for 60 s	1. Silane-containing adhesive agent 2. Silane-containing adhesive agent 3. Silane coupling agent	1. Clearfil Universal Bond Quick 2. Scotchbond Universal Adhesive 3. Optibond All in One	1. Panavia V5 2. RelyX Ultimate 3. NX3 Nexus
4	Gailani et al., 2021 [47]	Lava Ultimate, 3M ESPE	50 μm Al_2_O_3_ 60 s	No etching	Silane Rely X Ceramic primer	8 adhesives	Corresponding resin luting cements according to the manufacturers
5	Sag and Bektas, 2020 [48]	Lava Ultimate, 3M ESPE	-	37% phosphoric acid Both self-etch and etchandrinse	-	Single Bond Universal Adhesive	1. RelyX Ultimate Clicker 2. RelyXUnicem
6	Rozan et al., 2020 [49]	CerasmartGC	50 μm Al_2_O_3_ 10 s	37% phosphoric acid	1. Adhesive with silane 2. G-Multi Primer 3. Clearfil Ceramic Primer Plus	1. Scotchbond Universal Adhesive 2. G-Premio Bond, and 3.G-Premio Bond DCA	1. RelyX Ultimate 2. G-CEMLinkForce 3. Panavia V5
7	Ishi et al., 2017 [50]	1. Lava Ultimate, 3M ESPE 2. VITA Enamic	-	32% phosphoric acid	Adhesive with silane	Scotchbond Universal Adhesive	RelyX Ultimate
